# Engineering Docetaxel Micelles for Enhanced Cancer Therapy Through Intermolecular Forces

**DOI:** 10.3390/bioengineering11111078

**Published:** 2024-10-28

**Authors:** Hao Wang, Feirong Gong, Jiajie Liu, Lanlan Xiang, Yanfen Hu, Wenchen Che, Ran Li, Sisi Yang, Qixin Zhuang, Xin Teng

**Affiliations:** National Material Experimental Teaching Demonstration Center, East China University of Science and Technology, 130 Meilong Road, Shanghai 200237, Chinaqxzhuang@ecust.edu.cn (Q.Z.)

**Keywords:** docetaxel, nanomicelles, drug delivery, antitumor, intermolecular force

## Abstract

Docetaxel has exhibited excellent therapeutic effects in cancer treatment; however, its hydrophobicity, short blood circulation time, and high blood toxicity restrict its clinical application. The use of mPEG-PLA micelles to deliver docetaxel into the body has been verified as an effective approach to enhance its therapeutic efficacy. However, mPEG-PLA micelles are easily disassembled in the bloodstream, which can easily lead to premature drug release. To broaden the application scenarios of mPEG PLA micelles, we utilized the π–π stacking effect as an intermolecular force to design a novel mPEG-PLA-Lys(Fmoc) micelle to enhance the blood stability and permeability of drug-loaded micelles. The result showed that drug-loaded micelles for injection did not alter the tissue selectivity of docetaxel. Intravenous injection of the micelles in nude mice showed better antitumor efficacy than docetaxel injection and tumor recurrence rate is 0%, which is significantly lower than that of docetaxel injection (100%). The micelles designed by this research institute are anticipated to improve the clinical therapeutic effect of docetaxel.

## 1. Introduction

Docetaxel is a second-generation derivative of paclitaxel, which is widely used in the therapy of advanced breast cancer, non-small cell lung cancer, and ovarian cancer by preventing microtubule depolymerization and inducing cell apoptosis [1,2,3,4]. Docetaxel is mainly delivered via intravenous injection, but its solubility in water is extremely poor, which makes it prone to recognition and clearance by the reticuloendothelial system (RES) in the bloodstream [5]. This results in docetaxel being less readily absorbed by tumors, leading to poor antitumor efficacy and strong side effects.

Currently, the only docetaxel formulation available on the market is the docetaxel injection. The combination of docetaxel with liposomes and albumin has shown poor efficacy and has not achieved satisfactory clinical outcomes. In order to improve the clinical treatment effect of docetaxel, many studies have focused on nanomicelle drug delivery systems. Nanomedicine delivery systems are an effective approach to improving drug bioavailability. Nanocarriers include liposomes, vesicles, micelles, and others. These nanocarriers can enhance the permeability of formulations to target sites, as well as improve therapeutic efficacy and safety. For instance, they can help treat brain diseases including psychiatric and neurodegenerative disorders, where drugs previously struggled to penetrate the blood–brain barrier [6,7,8]. Additionally, nanocarriers can enhance the permeability of formulations at tumor sites [9]. Methoxy-Poly(ethylene glycol)-Poly(lactide) (mPEG-PLA) copolymer is currently the most widely used nanomicelle carrier [10,11]. Due to its amphiphilic structure, it can encapsulate insoluble drugs in hydrophobic cores to enhance their water solubility, and it simultaneously possesses high biocompatibility [12,13,14,15]. However, mPEG-PLA has poor stability in the bloodstream and is prone to disintegration, leading to the early release of drugs [16]. To address this issue, most previous research has focused on modifying mPEG-PLA to enhance its stability in the bloodstream and improve its interaction with drugs, thereby prolonging its circulation time. By grafting different functional groups onto the PLA segment, drug-loaded micelles can more stably exist in blood, and they can also have targeted drug delivery capabilities [17,18].

Enhancing the intermolecular interaction between the carrier and the drug is a good approach for enhancing the stability of drug-loaded micelles. The presence of multiple benzene rings in the structure of docetaxel provides a research basis for enhancing π–π conjugation interactions. Using this approach, researchers have significantly enhanced the stability of drug-loaded micelles in the bloodstream [19,20].

Fmoc (Fluorenylmethoxycarbonyl) has shown good performance in previous research because it can enhance the intermolecular interactions between the drug and the carrier [21,22]. The fluorene ring structure of Fmoc can not only form π–π conjugation with drugs but it can also facilitate this interaction between fluorene rings [23,24]. Fmoc is typically used as a protective group for peptides and amino acids. Considering the in vivo safety of micelles, non-toxic functional groups must be used for the modification of mPEG-PLA. Amino acids are a common group of substances in the human body and can be used in chemical treatment [25]. Amino acids protected by Fmoc perfectly meet the requirements for modification. Lysine is one of the essential amino acids that must be ingested but cannot be synthesized by the human body [26]. Amino acids that have a positive charge, such as arginine and lysine, are often used to facilitate the transmembrane transport of nanoparticles [27]. Previous research has demonstrated the safety and reliability of lysine in drug delivery systems [28,29,30,31]. Therefore, it is feasible to use both Fmoc and lysine for drug delivery.

**Figure 1:**
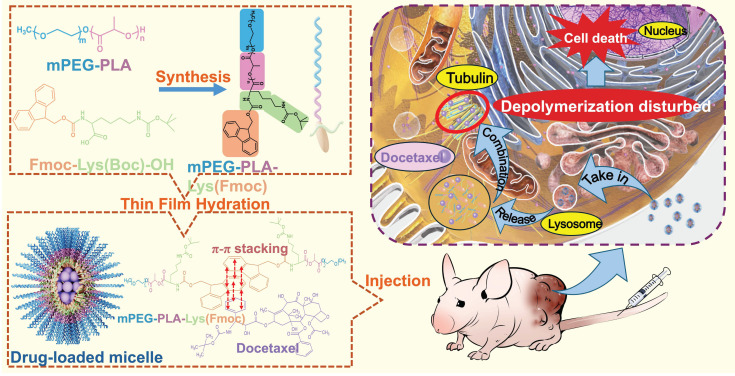
The formation process of the mPEG-PLA-Lys(Fmoc) and the intracellular mechanism.

This study has designed a novel mPEG-PLA-Lys(Fmoc) carrier that encapsulates docetaxel. The safety and anti-tumor effects of the carrier were comprehensively evaluated through in vitro characterization and animal experiments, as shown in Figure 1. Compared with docetaxel injection, the result showed better antitumor efficacy at the dosage of 20 mg/kg, and the recurrence rate was 0%, while docetaxel injection was 100%. The tissue distribution results indicate that the docetaxel-loaded micelles did not alter the distribution characteristics of docetaxel in different tissues. Pathological tissue sections, after acute and long-term toxicity experiments in beagle dogs and SD rats, also showed lower organ toxicity. The drug-loaded micelles designed by our study are expected to improve the side effects during the treatment of docetaxel.

## 2. Materials and Methods

### 2.1. Materials

Docetaxel (DTX) with a purity of >99.5%, poly(ethylene glycol)-poly(lactide) (mPEG-PLA, Mn = 2700, Mn(mPEG):Mn(PLA) = 3:1), pivaloyl chloride, and 4-Pyrrolidinopyridine (4-Py) were purchased from J&K Scientific Ltd. (Beijing, China). Fmoc-Lys(Boc)-OH was purchased from GL Biochem (Shanghai) Ltd. (Shanghai, China) with a purity of >99.0%. Triethylamine (TEA) was purchased from Shanghai Macklin Biochemical Technology Co., Ltd. (Shanghai, China) with a purity of >99.5%. Tetrahydrofuran (THF) was purchased from Shanghai Titan Scientific Co. Ltd. (Shanghai, China) with a purity of >99.0%. Ethanol was purchased from Sinopharm Chemical Reagent Co. Ltd. (Shanghai, China) with a purity of >99.5%. Ethyl ether was purchased from Shanghai Lingfeng Chemical Reagent Co. Ltd. (Shanghai, China) with a purity of >99.5%.

### 2.2. Cells and Animals

The human breast cancer cell line MCF-7 was purchased from the Evaluation Center of New Drug of Shandong Academy of Pharmaceutical Sciences (Jinan, China). Male and female Sprague-Dawley rats of specific pathogen-free (SPF) grade, weighing 180–250 g, were obtained from Shanghai Sippe-Bk Lab Animal Co., Ltd. (Shanghai, China). Male and female Beagle dogs of common grade, weighing 8.0–9.2 kg, were obtained from Jia’an Experimental Animal Breeding Co., Ltd. (Zhejiang, China). Female nude mice of SPF grade, weighing 15–17 g, were obtained from Beijing Vital River Laboratory Animal Technology Co., Ltd. (Beijing, China). Prior to the experiments, all animals were housed for 5–7 days in a room maintained at 20 ± 6 °C with 40–70% humidity and a controlled 12-h light–dark cycle. Male and Female ICR mice of SPF grade, weighing 16–18 g, were obtained from Shanghai Sippe-Bk Lab Animal Co., Ltd. (Shanghai, China). All animals had access to water and commercial laboratory complete food. All animal procedures were conducted in accordance with the protocol approved by the Evaluation Center of New Drug of Shandong Academy of Pharmaceutical Sciences (Jinan, China).

All animals were housed, experimented on, and euthanized in accordance with the China’s code of ethics for laboratory animals [32].

### 2.3. Synthesis of mPEG-PLA-Lys(Fmoc)

The synthesis process of mPEG-PLA-Lys(Fmoc) refers to the published papers of the author [33]. Fmoc-Lys(Boc)-OH (7.03 g, 15 mmol) and TEA (2.08 mL, 15 mmol) were dissolved in 50 mL of THF in an oven-dried flask and cooled to −10 °C. Then, 1.83 mL of pivaloyl chloride (15 mmol) was added, and the mixture was stirred in magnetic stirring at 0 °C for 2 h and then at room temperature for 1 h. After the reaction was complete, the mixture was filtered to remove insoluble substances. The filter residue was washed with a small amount of tetrahydrofuran, and the filtrate was combined. Vacuum rotary evaporation at 40 °C was used to remove the solvents. The product in the flask should be a colorless viscous liquid, which is then dissolved in 20 mL of dichloromethane.

The solution was added to 60 mL of dichloromethane containing 12 g of mPEG-PLA (molecular weight 2700), TEA (2.08 mL), and 4-Py (0.22 g). The mixture was stirred via magnetic stirring at 0 °C for 1 h, and then at room temperature for 36 h. Vacuum rotary evaporation at 30 °C was used to remove the solvents. After evaporation, 100 mL of ethanol was added to dissolve the residue, and the temperature was immediately raised to 45–50 °C. After dissolution, the mixture was immediately placed in an oil bath pre-cooled to −20 °C for magnetic stirring. After 10 min, crystal precipitation occurred. The mixture was filtered by vacuum filtration three times, and each time, the residue was immediately placed into cold ethanol (−20 °C). After the third filtration, the residue was put into cold ethyl ether (−20 °C). After filtration and drying under vacuum at room temperature for 48 h, mPEG-PLA-Lys(Fmoc) should be obtained as a white solid.

### 2.4. Characterization

The chemical structure of mPEG-PLA-Lys(Fmoc) was confirmed by proton nuclear magnetic resonance (^1^H NMR) spectroscopy, conducted in deuterated chloroform (CDCl_3_) at 25 °C using a Bruker NMR spectrometer (Avance III, 500 MHz, Billerica, MA, USA).

The morphology of both docetaxel-loaded and blank micelles was observed using a JEM-1800 transmission electron microscope (TEM, JEOL, Tokyo, Japan). A pipette was used to transfer a small amount of the sample onto the copper grid. The excess sample was removed to ensure a thin layer covers the grid. Then, the sample on the copper grid was stained with phosphotungstic acid reagent. After removing the excess staining agent, it was allowed to air dry. Subsequently, TEM images of the sample were captured.

The average diameter and size distribution of the micelles were determined by dynamic light scattering (DLS, Zetasizer Nano-ZS, Malvern Instruments, Malvern, UK). A pipette was used to transfer 1 mL of the sample into a four-way quartz cuvette, it was placed in the instrument, and the measurement mode with 3 cycles in Zetasizer Software (version 7.13) was selected.

Docetaxel concentrations in the micelle dispersions were quantified at 25 °C with an Agilent 1220 high-performance liquid chromatography (HPLC) system (Agilent Technologies, Santa Clara, CA, USA). The eluent was a mixture of acetonitrile and water (75/25), and the micelle dispersion was diluted with acetonitrile, filtered through a polyvinylidene fluoride (PVDF) filter, and then passed through an SB-C18 chromatographic column at a flow rate of 1 mL/min.

### 2.5. Preparation and Stability Evaluation of mPEG-PLA-Lys(Fmoc)/DTX

Docetaxel-loaded micelles (mPEG-PLA-Lys(Fmoc)/DTX) were prepared using the solid dispersion and thin film hydration technique. In this process, a mixture of 10 mg of docetaxel and 190 mg of mPEG-PLA-Lys(Fmoc) was dissolved in 5 mL of ethyl acetate at 40 °C. The solvent was then gradually evaporated under vacuum to form a thin film, which was subsequently hydrated with 5 mL of ultrapure water. The resulting micellar dispersion was filtered through a 0.22 μm PVDF filter to eliminate any insoluble impurities and large particles. This formulation typically consisted of 5% docetaxel and 95% mPEG-PLA-Lys(Fmoc).

The encapsulation efficiency and loading capacity were calculated using Equations (1) and (2), respectively. The drug content in micelles was determined from the docetaxel concentration in the dispersions, while the weight of the initial drug included free docetaxel that was not encapsulated, which was removed prior to HPLC analysis.

(1) $$\mathrm{Encapsulation}\;\mathrm{efficiency}=\frac{\mathrm{weight}\;\mathrm{of}\;\mathrm{drug}\;\mathrm{in}\;\mathrm{micelles}}{\mathrm{weight}\;\mathrm{of}\;\mathrm{the}\;\mathrm{initial}\;\mathrm{drug}}\times 100\%$$

(2) $$\mathrm{Loading}\;\mathrm{capacity}=\frac{\mathrm{weight}\;\mathrm{of}\;\mathrm{drug}\;\mathrm{in}\;\mathrm{micelles}}{\mathrm{weight}\;\mathrm{of}\;\mathrm{micelles}\;\mathrm{and}\;\mathrm{drug}}\times 100\%$$

In vitro stability was evaluated by testing diameters after 21 days of standing at 8 °C. The sample was placed in a vial, set against a black background, and photographed using the rear camera of a smartphone; meanwhile, photos were taken every 7 days to observe if there is any docetaxel precipitation.

### 2.6. Pharmacokinetics

Eight beagle dogs (four males and four females) were randomly divided into two groups, each consisting of two males and two females. The experimental group was injected with mPEG-PLA-Lys(Fmoc)/DTX, while the positive control group was injected with docetaxel injection. After intravenous administration of both docetaxel formulations at a dose of 45 mg/m^2^, 1.2 mL of blood was drawn from the vein at specified time points. The blood samples were centrifuged for 5 min to obtain plasma, and the docetaxel concentrations in the plasma were analyzed using a TSQ Quantum Access triple quadrupole liquid chromatography-mass spectrometry system (Thermo Fisher Scientific Inc., Waltham, MA, USA).

In a separate experiment, BALB/c mice were intravenously injected with 10 mg/kg of drug-loaded micelles and docetaxel injections. Blood, heart, liver, spleen, lung, kidney, stomach, and brain tissues were collected at 30, 60, 180, and 360 min post-administration for concentration analysis via LC-MS. The chromatographic conditions were as follows: an Agilent Eclipse Plus C18 column (150 mm × 2.1 mm, 5 μm) and an Agilent Eclipse Plus C18 guard column (12.5 mm × 2.1 mm, 5 μm) were used, with the column temperature maintained at 30 °C. The mobile phase consisted of a methanol–water gradient: 0–3 min, 90% water, and 10% methanol; 3–4 min, a gradient shift to 10% water and 90% methanol; 4–9 min, 10% water and 90% methanol; and 9–10 min, returning to 90% water and 10% methanol. The flow rate was set at 0.2 mL/min, with an injection volume of 10 μL. Mass spectrometry detection was conducted in the positive ion mode using SRM (Selected Reaction Monitoring).

### 2.7. In Vivo Antitumor Efficacy

The therapeutic efficacy of saline, blank excipient (mPEG-PLA-Lys(Fmoc)), docetaxel injection, and mPEG-PLA-Lys(Fmoc)/DTX was assessed in nude mice bearing xenografts of the human breast cancer cell line MCF-7. When the tumor volume reached approximately 160 mm^3^, the mice were separated by gender and further divided into six groups per gender (6 mice per group). The groups were treated with high-dose (20 mg/kg), medium-dose (10 mg/kg), and low-dose (5 mg/kg) mPEG-PLA-Lys(Fmoc)/DTX, administered every ten days, alongside comparison groups receiving docetaxel injection (20 mg/kg), physiological saline, or blank excipient. All of the dosing volumes were 10 mL/kg. After the initial treatment, the mice were monitored over a 30-day period for changes in body weight and tumor size (length and width). Mice were euthanized according to animal welfare guidelines if the tumor volume exceeded 2000 mm^3^. Tumor volume was calculated using the following equation:

$$V=\frac{1}{2}\mathrm{length}\times {\mathrm{width}}^{2}$$

### 2.8. Statistical Analysis

Unless otherwise noted, all values in this study represent the means of at least three independent experiments, with error bars indicating the standard deviations. Statistical differences between treatment groups were evaluated using one-way ANOVA followed by Tukey’s post hoc test for parametric data, or a nonparametric test such as the Mann–Whitney U test, where applicable. All analyses were performed using SPSS software (version 24.0). A *p*-value of less than 0.05 was deemed statistically significant.

## 3. Results and Discussion

### 3.1. Synthesis and Characterization of mPEG-PLA-Lys(Fmoc)

**Figure 2:**
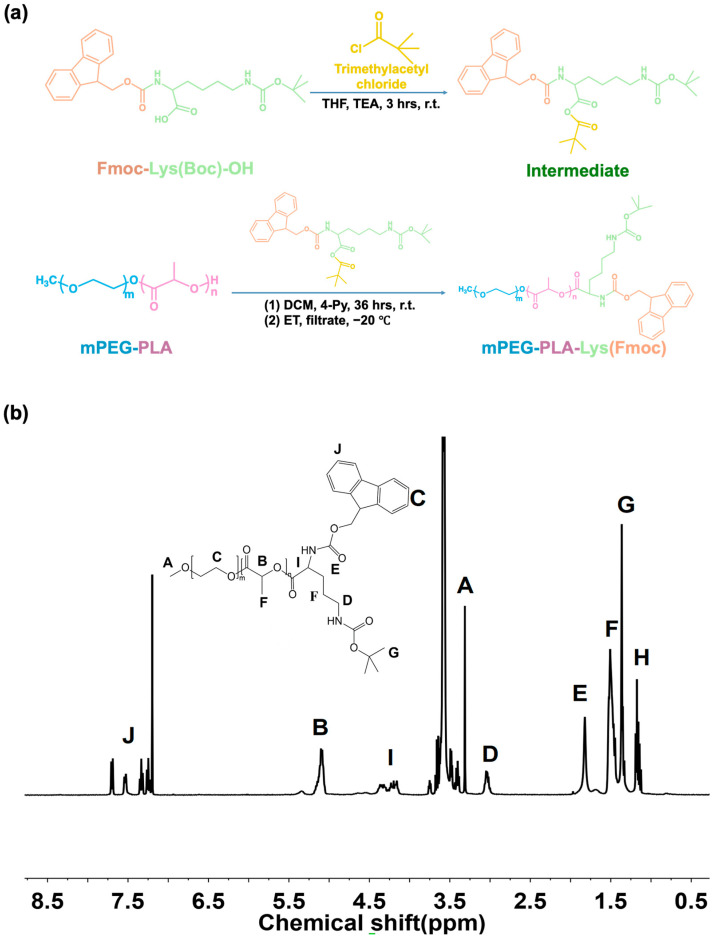
(**a**) The synthesis of mPEG-PLA-Lys(Fmoc). (**b**) ^1^H NMR spectrum of mPEG-PLA-Lys(Fmoc).

We controlled the synthetic method precisely at every step, leading to a well-defined structure of the resulting dendritic Lys(Fmoc) functionalized block copolymer. The detailed synthetic route is illustrated in Figure 2a.

The first step involved synthesizing the Lys(Fmoc) midbody via the reaction between the carboxyl group of Fmoc-Lys(Boc)-OH and acyl chloride group of pivaloyl chloride, generating an anhydride structure. The purpose of this step was to improve the reactivity of Fmoc-Lys(Boc)-OH. The second step involved synthesizing mPEG-PLA-Lys(Fmoc) via the reaction between mPEG-PLA and Lys(Fmoc) midbodies. In this research, the mPEG-PLA used was a pre-synthesized product. The number-average molecular weight (Mn) of the mPEG block was 2000 g/mol and that of the PLA block was 700 g/mol. Due to the high reactivity of the anhydride group and the catalytic effect of 4-Py catalyst, this step proceeded smoothly. As shown in the ^1^H NMR spectrum of mPEG-PLA-Lys(Fmoc) (Figure 2b), there are 10 distinct characteristic peaks. The chemical shifts for the corresponding peaks are as follows: point A has a chemical shift of δ = 3.31 ppm, point B has a chemical shift of δ = 5.09 ppm, point C has a chemical shift of δ = 3.58 ppm, point D has a chemical shift of δ = 3.03 ppm, point E has a chemical shift of δ = 1.82 ppm, point F has a chemical shift of δ = 1.48 ppm, point G has a chemical shift of δ = 1.36 ppm, point I has a chemical shift of δ = 4.25 ppm, and multiple chemical shifts between δ = 7.25 and 7.75 ppm at point J correspond to the protons on the aromatic ring of the Fmoc group. No extraneous peaks were observed, indicating a grafting efficiency of 100%. The result showed that all reactions were successfully carried out.

### 3.2. Micelle Formation

**Figure 3:**
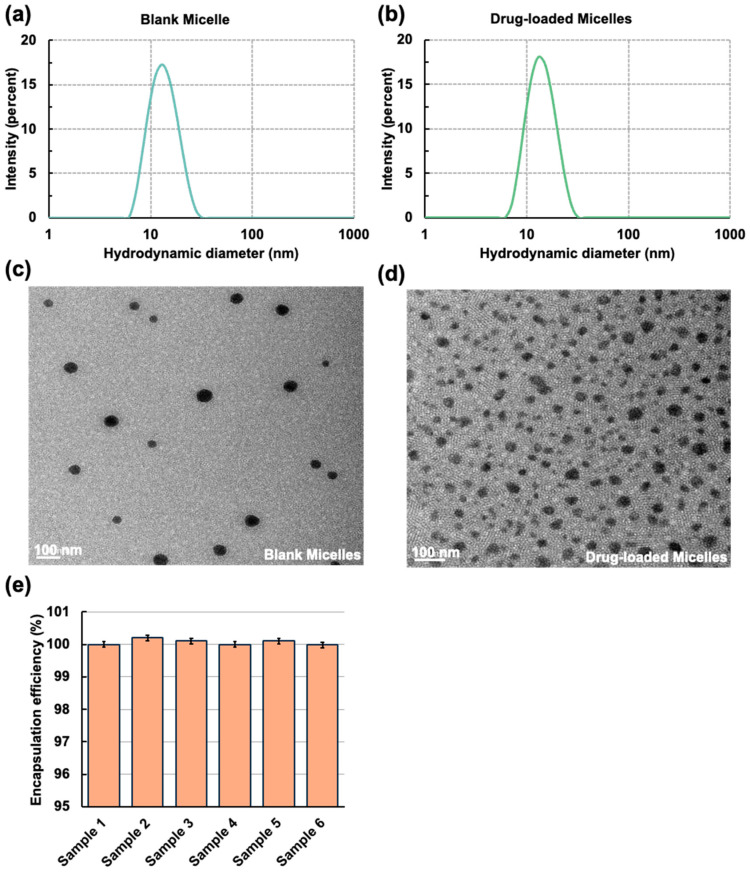
(**a**) Particle size distribution of blank micelles. (**b**) Particle size distribution of drug-loaded micelles. (**c**) TEM image of blank micelles. (**d**) TEM image of DTX-loaded micelles. (**e**) Encapsulation efficiency of preparation after the solid dispersion-thin film hydration method. The data are expressed as mean ± standard error, *n* = 3.

The drug-loaded micelles (mPEG-PLA-Lys(Fmoc)/DTX) were prepared using a solid dispersion-thin film hydration technique, which is highly scalable for industrial production. During this process, the amphiphilic mPEG-PLA-Lys(Fmoc) self-assembled into spherical micelles. The empty micelles had an average diameter of 13.71 nm with a polydispersity index (PDI) of 0.20, as measured by DLS (Figure 3a). The DTX-loaded micelles had an average diameter of 14.57 nm with a PDI of 0.1339 (Figure 3b). TEM images showed that both empty and drug-loaded micelles successfully formed spherical structures, consistent with expectations (Figure 3c,d). After filtering the sample with the 0.22 μm membrane, HPLC measurements showed that the loading efficiency was nearly 100%. Due to the extremely low solubility of free drug molecules, a small number of unsealed drugs were filtered out, and the encapsulation efficiency of drugs in the final product was close to 100%. Docetaxel is highly soluble in organic solvents like acetonitrile, ethanol, and ethyl acetate, so the solvent’s impact on encapsulation efficiency is minimal. Assuming no errors, the theoretical encapsulation efficiency was extremely close to 100% (Figure 3e).

### 3.3. In Vitro Stability

**Figure 4:**
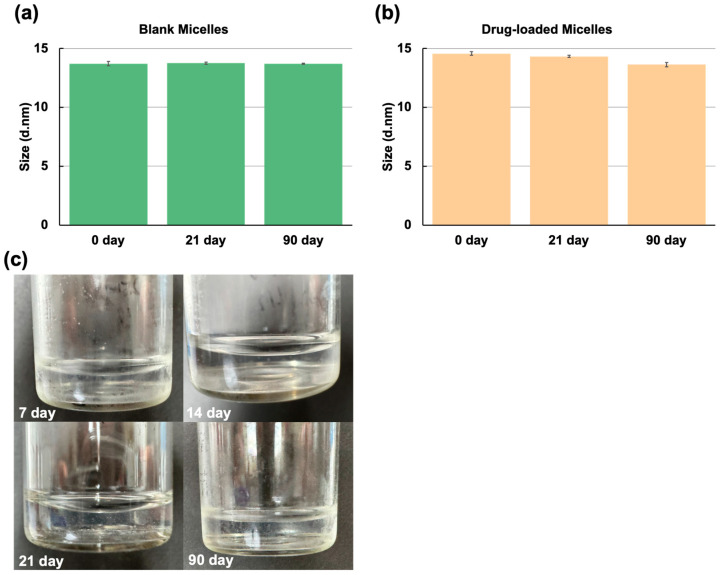
(**a**) Size variation in blank micelles. The data are expressed as mean ± standard error, *n* = 3. (**b**) Size variation in drug-loaded micelles. The data are expressed as mean ± standard error, *n* = 3. (**c**) Photographs of drug-loaded micelles kept at 8 °C on day 7, day 14, day 21, and day 90.

The stability of mPEG-PLA-Lys(Fmoc)/DTX was investigated over a period of 21 days by monitoring appearance status and changes in diameters and PDI. After storage, the same measurements were used to compare differences from the first day. A photo was taken of mPEG-PLA-Lys(Fmoc)/DTX stored at 8 °C during the experiment, and the diameter was recorded as measured by DLS. The result demonstrated that mPEG-PLA-Lys(Fmoc)/DTX exhibited excellent colloidal stability (Figure 4). Ordinary mPEG-PLA micelle solutions form drug precipitates within 6 h at room temperature due to the weak interactions between the PLA segments and the drug, which easily leads to drug release and precipitation. However, Fmoc micelles are much more stable at room temperature, undoubtedly due to the stronger interactions between Fmoc and the drug.

### 3.4. Pharmacokinetics Result

The free drug is known to be rapidly eliminated and metabolized by the liver; thus, the in vivo stability is quite important for drug delivery.

**Figure 5:**
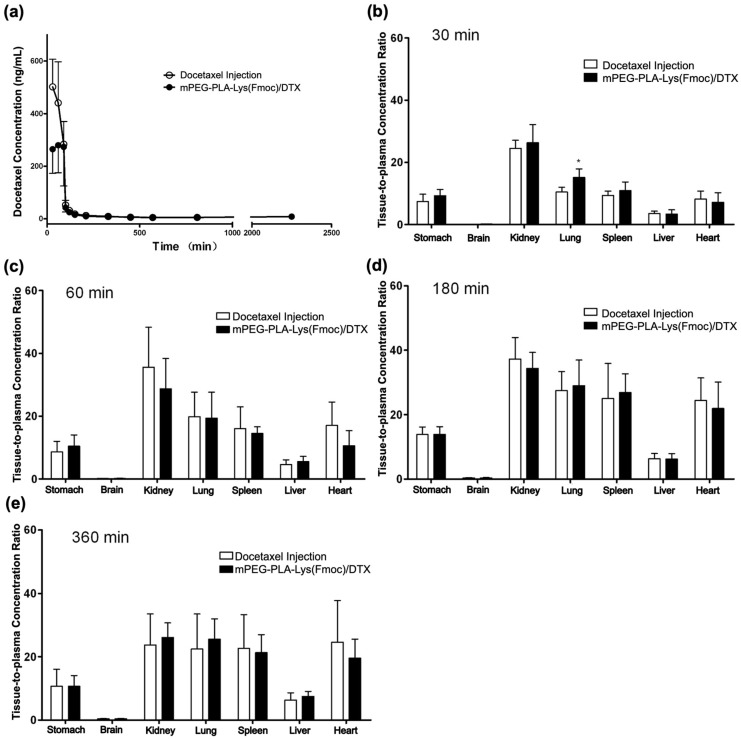
(**a**) Plasma docetaxel concentration–time curves after i.v. administration of docetaxel injection and mPEG-PLA-Lys(Fmoc)/DTX in Beagle dogs with a single dosage of 45 mg/m^2^. (**b**–**e**) Tissue-to-plasma concentration ratios at various times following i.v. administration of docetaxel injection and in mPEG-PLA-Lys(Fmoc)/DTX in mice with the single dosage of 10 mg/kg (*, *p* < 0.05. The data are expressed as mean ± standard error, *n* = 3.

The recommended dosage of Taxotere^®^ (docetaxel injection), as stated in the prescribing information, is 75 mg/m^2^ in the human body. Considering species differences, we selected a dosage of 45 mg/m^2^ for Beagle dogs to conduct pharmacokinetic studies of docetaxel injection and mPEG-PLA-Lys(Fmoc)/DTX. Docetaxel concentration–time curves in the plasma of beagle dogs are demonstrated in Figure 5a. It can be clearly observed that the initial concentration of the drug-loaded micelles is lower than the docetaxel injection because most of the drug-loaded micelles enter the tissue. The retention time in the bloodstream is significantly longer than that of the docetaxel injection, which means the stability in blood and permeability to the tissue of the drug-loaded micelles are superior to docetaxel injection. The primary pharmacokinetic parameters of the formulations were determined using non-compartmental analysis, as detailed in Table 1. The result showed that the metabolism of both docetaxel injection and mPEG-PLA-Lys(Fmoc)/DTX in Beagle dogs conform to the three-compartment model. The C_max_ and AUC_(0-t)_ of mPEG-PLA-Lys(Fmoc)/DTX were significantly lower than that of the docetaxel injection under the same dosage, which indicated that drug-loaded micelles penetrate tissues more easily, thus making lower blood toxicity. Other pharmacokinetic parameters, such as AUC_(0-∞)_, t_1/2_, and CL, were compared and showed no significant difference. In summary, the pharmacokinetics of mPEG-PLA-Lys(Fmoc)/DTX in Beagle dogs is much better than docetaxel injection.

The drug distribution trends of the two formulations in the blood, heart, liver, spleen, lung, kidney, stomach, and brain at 30, 60, 180, and 360 min were similar, with the highest concentration observed in the kidneys and the lowest in the brain (Figure 5b–e). Except for certain tissues at specific time points, there were no significant differences in the tissue-to-plasma concentration ratios between the two formulations. Therefore, the drug-loaded micelles for injection did not alter the tissue selectivity of docetaxel.

### 3.5. In Vivo Cytotoxicity

**Figure 6:**
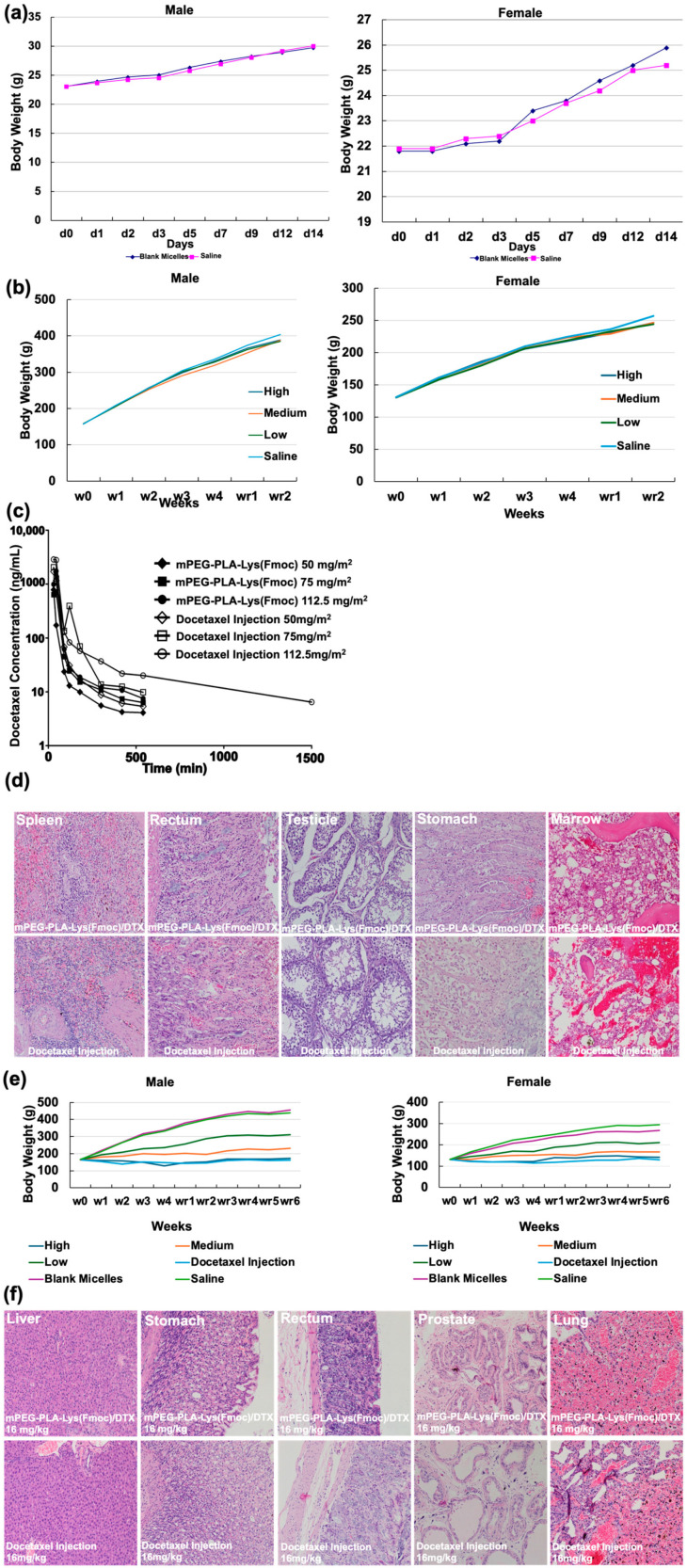
(**a**) Body weight change (g) of male and female IRC mice after i.v. administration of blank micelles at the max dosage of 6080 mg/kg in acute experiments. (**b**) Body weight change (g) in male and female SD rats after i.v. administrated blank micelles at the max dosage of 2280 mg/kg in a long-term experiment. (**c**) Plasma docetaxel concentration–time curves after i.v. administration of docetaxel injection and mPEG-PLA-Lys(Fmoc)/DTX in Beagle dogs with a single dosage of 50 mg/kg, 75 mg/kg, and 112.5 mg/kg in acute experiments. (**d**) Pathological section photographs of main organs of Beagle dogs after i.v. administration of docetaxel injection and DTX-loaded micelles in acute experiments. (**e**) Body weight change (g) in male and female SD rats after various treatments. (**f**) Pathological section photographs of the liver, stomach, rectum, and prostate of SD rats after i.v. administration of docetaxel injection (16 mg/kg) and DTX-loaded micelles (16 mg/kg) in long-term experiments.

Micelles used as carriers must not be toxic to humans. Acute and long-term toxicity experiments were performed on blank micelles (mPEG-PLA-Lys(Fmoc)) using ICR mice for acute testing and SD rats for long-term studies, with body weight changes recorded. The results showed that in both acute and long-term toxicity experiments, no significant appearance or behavioral abnormalities were observed at the max dosage of 6080 mg/kg (acute) and 2280 mg/kg (long-term) in both male and female groups. Furthermore, similar changes in body weight also confirmed these findings (Figure 6a,b).

Although the safety of the carrier is important, the drugs it carries are what truly have therapeutic effects. The harm of drugs to the human body cannot be ignored; therefore, it is necessary to determine the safe dosage of the drug. The results of the acute toxicity experiments of mPEG-PLA-Lys(Fmoc)/DTX after intravenous administration in Beagle dogs suggested that at dosages of 50 mg/m^2^, 75 mg/m^2^, and 112.5 mg/m^2^, dogs exhibited varying degrees of reduced activity, decreased food and water intake, and weight loss. All dogs died within a 21-day observation period at the dosage of 75 mg/m^2^ and 112.5 mg/m^2^. Under the conditions of this experiment, the approximate lethal dose of mPEG-PLA-Lys(Fmoc)/DTX is estimated to be between 50 and 75 mg/m^2^. Comparatively, the result of docetaxel injection after intravenous administration in Beagle dogs showed that at the dosage of 50 mg/m^2^, 75 mg/m^2^, and 112.5 mg/m^2^, in addition to causing struggles and unease in the experimental dogs, there were also varying degrees of congestion, skin laxity, wrinkles, and other allergic reactions in the face, ears, and eyelids. Other toxic reactions were similar to those of mPEG-PLA-Lys(Fmoc)/DTX. The approximate lethal dose and toxic target organs of the docetaxel injection were also the same as those of mPEG-PLA-Lys(Fmoc)/DTX. However, at the same dose, the exposure level of docetaxel injection was higher than that of mPEG-PLA-Lys(Fmoc)/DTX. The drug concentration–time curve and pathological tissue sections further confirmed these findings (Figure 6c,d).

The long-term toxicity experiment results of mPEG-PLA-Lys(Fmoc)/DTX after intravenous administration in SD rats suggested that when continuously injected at dosages of 4 mg/kg, 8 mg/kg, and 16 mg/kg for four weeks (once a week), the main symptoms during the entire experimental period were reduced activity, vertical hair, hair loss, emaciation, sparse hair cover, and tail peeling. Some individuals experienced swelling of the nasal wings, mouth, and back, as well as hunched backs. Near-death animals also exhibited decreased body temperature, near-death breathing, and a supine position. In the high-dose group, animal deaths occurred. The reaction increased with the prolongation of administration time, and the severity, duration, number, and type of animal reactions were positively correlated with the dosage. However, except for the tail peeling reaction, all other reactions could recover within six weeks of discontinuation. In comparison, except for much more severe symptoms, untoward reactions lasted until the end of the experiment after the intravenous administration of docetaxel injection (16 mg/kg) in SD rats at the same dosage. The changes in body weight and pathological tissue sections also confirmed these findings (Figure 6e,f).

All in all, the in vivo cytotoxicity experiment results showed that the side effects of mPEG-PLA-Lys(Fmoc)/DTX are fewer than those of the docetaxel injection.

### 3.6. Antitumor Efficacy

**Figure 7:**
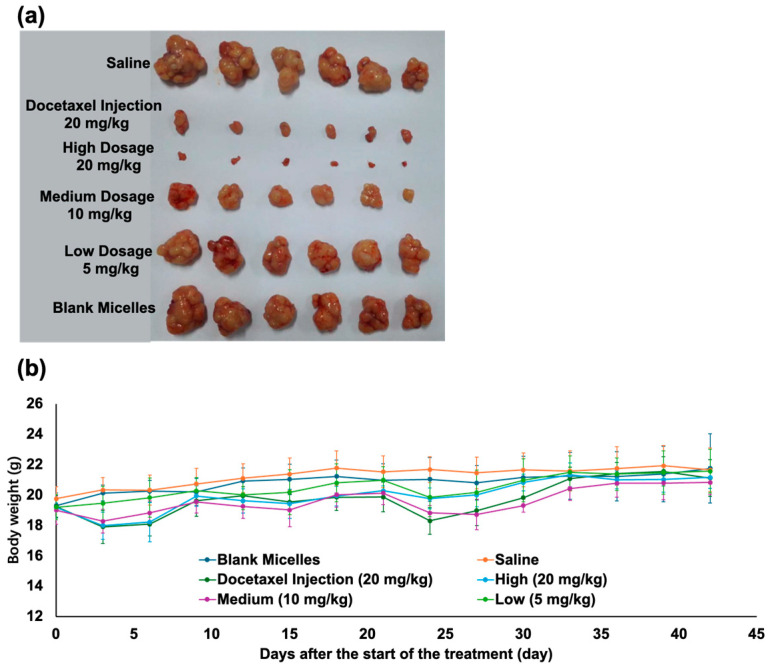
(**a**) Tumors were extracted from female mice euthanized at various time points. (**b**) Changes in body weight (g) of female mice under different treatments. The data are expressed as mean ± standard error, *n* = 3.

The in vivo antitumor efficacy of mPEG-PLA-Lys(Fmoc)/DTX was evaluated using a human breast cancer MCF-7 cell line xenograft in female nude mice. The initial tumor volume is about 160 mm^3^. In the low dosage (5 mg/kg) group of mPEG-PLA-Lys(Fmoc)/DTX, the result indicated poor antitumor efficacy, but as the dosage increased, the antitumor efficacy also increased. At the dosage of 20 mg/kg, drug-loaded micelles exhibited a better tumor suppression effect, with no tumor recurrence during the injection cycle (Figure 7a). In the antitumor experiment, the T/C of the drug-loaded micelles was 0.98%, while the docetaxel injection was 4.7% (Table 2), indicating that the drug-loaded micelles have a superior antitumor effect. We defined recurrence as the tumor volume growing to four times that before administration. Compared to the 100% tumor recurrence rate of docetaxel injection, the result was 0% for the high-dosage group of drug-loaded micelles. Both docetaxel injection and drug-loaded micelles caused body weight loss. As shown in Figure 6b, mice after i.v. injected with medium and high doses of drug-loaded micelles and docetaxel injection experienced weight loss within 3 days. Among them, docetaxel injection and high-dose drug-loaded micelles caused significant weight loss, with the high-dose micelles causing relatively less weight loss. Mice treated with docetaxel injection and drug-loaded micelles were observed in repeated cycles of weight loss and gain during the experiment. All of the mice gained weight after 25 days, and the body weight tended to stabilize.

In conclusion, mPEG-PLA-Lys(Fmoc)/DTX, at a dosage of 5 mg/kg, had no effect on inhibiting tumors, whereas, at 10 mg/kg, it had a certain effect. At 20 mg/kg, it showed better antitumor efficacy than the docetaxel injection at the same dosage (*p* < 0.05). The tumor recurrence rate showed a significant improvement compared to the docetaxel injection (100%) with a rate of 0%.

## 4. Conclusions

We designed and synthesized a mPEG-PLA-Lys(Fmoc) micelle for in vivo delivery of docetaxel and for evaluating its in vitro stability, in vivo cytotoxicity, pharmacokinetics, and antitumor efficacy against human breast cancer cells (MCF-7). The formulation, when loaded with mPEG-PLA-Lys(Fmoc), reduced adverse reactions and side effects of docetaxel following intravenous (i.v.) administration compared to the traditional docetaxel injection. The results demonstrated a high tumor inhibition rate and a low recurrence rate in terms of antitumor efficacy. Therefore, our micellar formulation is anticipated to offer a feasible and efficacious approach for the delivery of docetaxel, thereby enhancing cancer therapy and lowering side effects. In the future, we will continue to study possible micelle structures to further improve the safety of docetaxel in cancer treatment, such as passive and active targeting, and explore the feasibility of using micelles for traditional drug delivery.

## Figures and Tables

**Table 1:** Pharmacokinetic parameters after i.v. administration of docetaxel injection and mPEG-PLA-Lys(Fmoc)/DTX in beagle dogs with a single dosage of 45 mg/m^2^.

Parameters	Group	
	Docetaxel Injection	mPEG-PLA-Lys(Fmoc)/DTX
AUC_(0–t)_, μg/L·min	(4.2 ± 0.8) × 10^4^	(3.2 ± 1.1) × 10^4^
AUC_(0–∞)_, μg/L·min	(4.3 ± 0.8) × 10^4^	(4.1 ± 2.1) × 10^4^
MRT_(0–t)_, min	(8.5 ± 2.3) × 10	(1.6 ± 1.6) × 10^2^
MRT_(0–∞)_, min	(9.8 ± 2.5) × 10	(1.1 ± 1.9) × 10^3^
t_1/2z_, min	(1.7 ± 0.8) × 10^2^	(1.2 ± 1.9) × 10^3^
V_z_, L/m^2^	(2.5 ± 0.9) × 10^2^	(1.4 ± 1.6) × 10^3^
CL_z_, L/min/m^2^	1.1 ± 0.2	1.5 ± 1.1
C_max_, μg/L	(5.3 ± 1.0) × 10^2^	(3.3 ± 1.2) × 10^2^ **

**, *p* < 0.01.

**Table 2:** The effects of different administration groups on the growth inhibition rate of human MCF-7 xenograft tumors in female nude mice.

Group	Frequency (Time)	Volume (mm^3^)		T/C (%)	Tumor Weight (g)	Recurrence Rate (%)	Antitumor Rate (%)
		Initial	Final				
Saline	3	(1.6 ± 0.5) × 10^2^	(2.2 ± 0.8) × 10^3^	--	2.1 ± 1.1	--	--
Docetaxel injection	3	(1.6 ± 0.4) × 10^2^	(1.0 ± 0.6) × 10^2^	4.70	0.10 ± 0.07	100 (6/6)	94.96
Low	3	(1.6 ± 0.5) × 10^2^	(1.7 ± 0.3) × 10^3^	77.81	1.7 ± 0.3	--	19.44
Mid	3	(1.6 ± 0.3) × 10^2^	(6.6 ± 2.8) × 10^2^	29.96	0.60 ± 0.03	--	72.25
High	3	(1.6 ± 0.2) × 10^2^	(2.1 ± 1.1) × 10	0.98	0.010 ± 0.007	0 (0/6)	99.27
Blank	3	(1.6 ± 0.6) × 10^2^	(2.1 ± 0.3) × 10^3^	97.03	2.0 ± 0.6	--	1.45

## Data Availability

The raw data supporting the conclusions of this article will be made available by the authors on request.

## References

[1] Vaishampayan U.N., Keessen M., Dreicer R., Heath E.I., Buchler T., Árkosy P.F., Csöszi T., Wiechno P., Kopyltsov E., Orlov S.V. (2024). A global phase II randomized trial comparing oral taxane ModraDoc006/r to intravenous docetaxel in metastatic castration resistant prostate cancer. Eur. J. Cancer.

[2] Dacos M., Immordino B., Diroff E., Sicard G., Kosta A., Rodallec A., Giacometti S., Ciccolini J., Fanciullino R. (2024). Pegylated liposome encapsulating docetaxel using microfluidic mixing technique: Process optimization and results in breast cancer models. Int. J. Pharm..

[3] Ya-Jung W., Jung-Jung T., Ming-Wei L., Ling-Ming T., Chih-Jung W. (2024). Revealing symptom profiles: A pre-post analysis of docetaxel therapy in individuals with breast cancer. Eur. J. Oncol. Nurs..

[4] Pisano C., Turco F., Arnaudo E., Fea E., Vanella P., Ruatta F., Filippi R., Brusa F., Prati V., Vana F. (2024). TEAM Study: Upfront Docetaxel Treatment in Patients With Metastatic Hormone-Sensitive Prostate Cancer: A Real-World, Multicenter, Retrospective Analysis. Clin. Genitourin. Cancer.

[5] Cai Y., Qi J., Lu Y., He H., Wu W. (2022). The in vivo fate of polymeric micelles. Adv. Drug Deliv. Rev..

[6] Pires P.C., Paiva-Santos A.C., Veiga F. (2023). Liposome-Derived Nanosystems for the Treatment of Behavioral and Neurodegenerative Diseases: The Promise of Niosomes, Transfersomes, and Ethosomes for Increased Brain Drug Bioavailability. Pharmaceuticals.

[7] Harini K., Alomar S.Y., Vajagathali M., Manoharadas S., Thirumalai A., Girigoswami K., Girigoswami A. (2024). Niosomal Bupropion: Exploring Therapeutic Frontiers through Behavioral Profiling. Pharmaceuticals.

[8] Pires P.C., Rodrigues M., Alves G., Santos A.O. (2022). Strategies to Improve Drug Strength in Nasal Preparations for Brain Delivery of Low Aqueous Solubility Drugs. Pharmaceutics.

[9] Thirumalai A., Girigoswami K., Pallavi P., Harini K., Gowtham P., Girigoswami A. (2023). Cancer therapy with iRGD as a tumor-penetrating peptide. Bull. Cancer.

[10] Sell M., Lopes A.R., Escudeiro M., Esteves B., Monteiro A.R., Trindade T., Cruz-Lopes L. (2023). Application of Nanoparticles in Cancer Treatment: A Concise Review. Nanomaterials.

[11] Yun W.S., Kim J., Lim D.-K., Kim D.-H., Jeon S.I., Kim K. (2023). Recent Studies and Progress in the Intratumoral Administration of Nano-Sized Drug Delivery Systems. Nanomaterials.

[12] Scaffaro R., Lopresti F., Marino A., Nostro A. (2018). Antimicrobial additives for poly (lactic acid) materials and their applications: Current state and perspectives. Appl. Microbiol. Biotechnol..

[13] He M., Zhang Z., Jiao Z., Yan M., Miao P., Wei Z., Leng X., Li Y., Fan J., Sun W. (2023). Redox-responsive phenyl-functionalized polylactide micelles for enhancing Ru complexes delivery and phototherapy. Chin. Chem. Lett..

[14] Repp L., Unterberger C.J., Ye Z., Feltenberger J.B., Swanson S.M., Marker P.C., Kwon G.S. (2021). Oligo(Lactic Acid)8-Docetaxel Prodrug-Loaded PEG-b-PLA Micelles for Prostate Cancer. Nanomaterials.

[15] Waris A., Ali A., Khan A.U., Asim M., Zamel D., Fatima K., Raziq A., Khan M.A., Akbar N., Baset A. (2022). Applications of Various Types of Nanomaterials for the Treatment of Neurological Disorders. Nanomaterials.

[16] Avgoustakis K., Beletsi A., Panagi Z., Klepetsanis P., Livaniou E., Evangelatos G., Ithakissios D. (2003). Effect of copolymer composition on the physicochemical characteristics, in vitro stability, and biodistribution of PLGA–mPEG nanoparticles. Int. J. Pharm..

[17] Ouyang C., Zhang W., Nie J., Yu L., Liu J., Ren L., Chen G. (2023). Nanoparticles with Active Targeting Ability and Acid Responsiveness for an Enhanced Antitumor Effect of Docetaxel. Biomacromolecules.

[18] Tariq I., Hassan H., Ali S., Raza S.A., Shah P.A., Ali M.Y., Tariq Z., Bakowsky U. (2024). Ameliorative Delivery of Docetaxel and Curcumin using PEG Decorated Lipomers: A Cutting-Edge In-Vitro/In-Vivo Appraisal. J. Drug Deliv. Sci. Technol..

[19] Pei Q., Jiang B., Hao D., Xie Z. (2023). Self-assembled nanoformulations of paclitaxel for enhanced cancer theranostics. Acta Pharm. Sin. B.

[20] Shi Y., Van Der Meel R., Theek B., Oude Blenke E., Pieters E.H., Fens M.H., Ehling J., Schiffelers R.M., Storm G., Van Nostrum C.F. (2015). Complete regression of xenograft tumors upon targeted delivery of paclitaxel via Π–Π stacking stabilized polymeric micelles. ACS Nano.

[21] Yang Z.-y., Zhong Y.-y., Zheng J., Liu Y., Li T., Hu E., Zhu X.-f., Ding R.-q., Wu Y., Zhang Y. (2021). Fmoc-amino acid-based hydrogel vehicle for delivery of amygdalin to perform neuroprotection. Smart Mater. Med..

[22] Ischakov R., Adler-Abramovich L., Buzhansky L., Shekhter T., Gazit E. (2013). Peptide-based hydrogel nanoparticles as effective drug delivery agents. Bioorg. Med. Chem..

[23] Zhang P., Huang Y., Liu H., Marquez R.T., Lu J., Zhao W., Zhang X., Gao X., Li J., Venkataramanan R. (2014). A PEG-Fmoc conjugate as a nanocarrier for paclitaxel. Biomaterials.

[24] Zhang P., Li J., Ghazwani M., Zhao W., Huang Y., Zhang X., Venkataramanan R., Li S. (2015). Effective co-delivery of doxorubicin and dasatinib using a PEG-Fmoc nanocarrier for combination cancer chemotherapy. Biomaterials.

[25] Toderascu L.I., Sima L.E., Orobeti S., Florian P.E., Icriverzi M., Maraloiu V.-A., Comanescu C., Iacob N., Kuncser V., Antohe I. (2023). Synthesis and Anti-Melanoma Activity of L-Cysteine-Coated Iron Oxide Nanoparticles Loaded with Doxorubicin. Nanomaterials.

[26] Gunarathne R., Guan X., Feng T., Zhao Y., Lu J. (2024). L-lysine dietary supplementation for childhood and adolescent growth: Promises and precautions. J. Adv. Res..

[27] Tian R., Wang H., Niu R., Ding D. (2015). Drug delivery with nanospherical supramolecular cell penetrating peptide–taxol conjugates containing a high drug loading. J. Colloid Interface Sci..

[28] Moghaddam S.V., Abedi F., Alizadeh E., Baradaran B., Annabi N., Akbarzadeh A., Davaran S. (2020). Lysine-embedded cellulose-based nanosystem for efficient dual-delivery of chemotherapeutics in combination cancer therapy. Carbohydr. Polym..

[29] Liu Y., Li J., Shao K., Huang R., Ye L., Lou J., Jiang C. (2010). A leptin derived 30-amino-acid peptide modified pegylated poly-L-lysine dendrigraft for brain targeted gene delivery. Biomaterials.

[30] Moghadam M.R., Karimi S., Namazi H. (2024). A targeted biosystem based on l-lysine coated GO@ rod-Cu (II) metal-organic frameworks for pH-controlled co-delivery of doxorubicin and curcumin. Food Biosci..

[31] Pang J., Zhuang B., Zhang L.-M. (2023). A co-carrier for plasmid DNA and curcumin delivery to treat pancreatic cancer via dendritic poly (l-lysine) modified amylose. Int. J. Biol. Macromol..

[32] (2022). Laboratory Animals—General Code of Animal Welfare.

[33] Qi D., Gong F., Teng X., Ma M., Wen H., Yuan W., Cheng Y., Lu C. (2017). Design and evaluation of mPEG-PLA micelles functionalized with drug-interactive domains as improved drug carriers for docetaxel delivery. J. Biomater. Sci. Polym. Ed..

